# Effectiveness, satisfaction and meaningfulness of a 6-step detection and prevention program for frail community-dwelling older adults: a mixed-method evaluation

**DOI:** 10.1186/s12877-022-03504-7

**Published:** 2022-12-14

**Authors:** Eva Dierckx, Daan Duppen, Sylvia Hoens, Lise Switsers, An-Sofie Smetcoren, Liesbeth De Donder, D-SCOPE

**Affiliations:** 1 Faculty of Psychology and Educational Sciences, Vrije Universiteit Brussel (VUB), Pleinlaan 2, 1050 Brussels, Belgium; 2 Psychiatric Hospital Alexianen Zorggroep Tienen, Tienen, Belgium; 3 Research Foundation Flanders (FWO), Brussels, Belgium; 4 Society and Ageing Research Lab, Brussels, Belgium

**Keywords:** Multidimensional frailty, Detection, Prevention, Risk factors, Warm referral, Follow up, Randomized controlled trial

## Abstract

**Background:**

As people age, the risk of becoming frail increases, often leading to negative outcomes and less well-being. Within the light of prevention, early detection and guidance to the right care and support is crucial. This study aimed to give an overview of the descriptive results of the D-SCOPE program and evaluate the process.

**Methods:**

The D-SCOPE program was developed as a detection and prevention program for frail community-dwelling older adults. The program creates a continuum of care and support, consisting of 6 steps: (1) Targeted case-finding using risk profiles for purposeful selection, (2) Preventive home-visit by an older volunteer, (3) Home visits by a professional, (4), Warm referral, (5) Monthly follow-up and (6) Long-term follow-up by home visit. The effectiveness of this program, in terms of satisfaction and meaningfulness, was studied quantitatively by means of a randomized controlled trial amongst 869 people with a frailty risk profile and qualitatively by 15 focus groups interviews.

**Results:**

The quantitative study revealed that 83.9% of the participants found the different home visits within the D-SCOPE program useful. The focus group interviews shed light on several issues and advantages: a more efficient case finding due to the applied risk factors for frailty, a more intensive tailor-made care and support due to the warm referral, the importance of both small-scaled and larger interventions based on the wishes irrespective of the state of frailty of the older persons, the focus on a strengths-based instead of a deficit-based approach and the follow up as being one of the greatest strengths of the project. However, to fully understand the benefits of the program a shift in mind from intervention to prevention is necessary.

**Conclusions:**

Our quantitative data show that most participants found the home visits meaningful and were satisfied with the intervention. The qualitative findings provided more insights into the experiences of the participants with the process. Based on these insights of the 6-step model of preventive home visits, municipalities and organizations can apply this model to carry out more targeted home visits.

**Trial registration:**

This trial was registered at ClinicalTrials.gov, on 30/05/2017, identifier: NCT03168204.

## Background

A systematic review and meta-analysis on frailty prevalence in 22 European countries showed a pooled prevalence rate of 12% among community-dwelling older people, when using the physical phenotype and 16% when using other frailty classification approaches [1]. While extensive research focuses on interventions on frailty [2], a recent review and meta-analysis concludes that interventions for frail community-dwelling older adults such as case management, information provision or technological interventions and more have no significant effect on adverse outcomes. The ones that do have an effect are custom made interventions [3]. Therefore, Hertogh (2013) proposed that we should not focus on combating frailty, but rather emphasize active anticipation of frailty and focus on detection and prevention [2]. However, it is still a challenge to detect frail, community-dwelling older adults [4].

In order to detect these older people, the Detection, Support and Care for Older people, Prevention and Empowerment (D-SCOPE) program was developed which is a multidimensional detection and prevention program for frail community-dwelling older adults. The D-SCOPE program creates a continuum of care and support for frail community-dwelling older adults and consists of 6 steps: (1) Targeted case-finding using risk profiles for purposeful selection, (2) Preventive home-visit by an older volunteer, (3) Home visit 2 by a professional, (4), Warm referral, (5) Monthly follow-up, (6) Long-term follow-up by home visit. The D-SCOPE program was implemented in three municipalities in Belgium to improve the detection of older people (at risk of) being or experiencing frailty and their guidance towards care and support. The D-SCOPE program addresses two main innovations or challenges. First, it operationalizes frailty as a multidimensional concept, as the complex interplay between physical, psychological, social, cognitive and environmental frailty dimensions [5, 6]. Second, while the vast majority of frailty research on outcomes for older persons focuses on adverse medical outcomes, such as mortality or hospitalization, this program focuses on well-being as primary outcomes, including life satisfaction, mastery and meaning in life [7], moving away from a merely deficit-based frailty approach towards a dynamic and strengths-based perspective [8].

The aim of the current paper is to give an overview of the descriptive results of the D-SCOPE program and evaluate the process, referring to the 6 steps, to determine the facilitating and hindering components when implementing the D-SCOPE program.

## Methods

### Study design

The D-SCOPE program was evaluated using a mixed method study with an exploratory design, starting with the quantitative data collection and analysis of a Randomized Controlled Trial, which has been conducted in three municipalities in Flanders (Belgium): Knokke-Heist, Ghent and Tienen. The quantitative part was followed with a qualitative part with stakeholders in the same municipalities. After combining the results of both studies, the program was modified. Community-dwelling people aged 60 years and over were included in the quantitative study. The two-wave interview survey was administered between 1 and 2017 and 30 June 2018 and at baseline participants were randomly selected from the census records in each municipality. The inclusion criteria were designed to target frail community-dwelling older people at risk of frailty, through risk profiles based on demographic characteristics such as age, gender, marital status, migration background, and having moved in the past 10 years. More information about the risk profiles can be found in Dury [4]. At baseline, 869 people with a frailty risk profile responded (Knokke-Heist: N = 293, Ghent = N = 299 and Tienen: N = 277). The follow-up survey was conducted six months later, and 540 respondents agreed to participate in the follow-up. People were excluded if, for any reason, they were not present at the address at the time of the baseline test period, if they were hospitalized, had moved to special housing, or when the respondent could not be reached after several trials. In advance, respondents received a letter with information; at the start of the survey respondents were informed by the interviewer and they received a letter for their general practitioner and (informal) caregivers. The Medical Ethics Committee of Vrije Universiteit approved the study (Reference number: B.U.N. 143,201,630,458). The trial was performed as planned. Additional information about the study design can be found in a published study protocol [9].

The qualitative data used in this study consisted of anonymized transcripts of 15 focus group interviews with a mean time of 75 min. Within one month after ending the D-SCOPE program, five different stakeholder groups were invited to the focus groups: older adults of the experimental group, older adults of the control group, informal caregivers, volunteers who participated in the preventive home visit 1 and professionals who organized and conducted the second home visits, referral, and follow-up. A focus group with each stakeholders group was conducted in each of the three municipalities, resulting in 15 focus groups. For each focus group between 2 and 9 participants were selected. Earlier research recommends focusing on a small number of participants when there is a high emotional involvement within the focus group or when the participants are experts [10]. In Spring 2018, the participants were purposively selected, to create a variation on the experience of diverse parts of the D-SCOPE program. Eligible older adults and informal caregivers were contacted by telephone by the contact person of the municipality about the purpose of the focus group and practical information. When they expressed an interest, they were invited to the focus group. All the volunteers and professionals were invited to a focus group in their municipality. The focus groups took place in the local service center of each municipality. In total, 74 people participated: 18 older adults from the experimental group, 14 older adults from the control group, 16 informal caregivers, 17 volunteers and 9 professionals. (See Table 1 for a detailed overview of the participants’ characteristics).

**Table 1:** Overview participants focus groups

Stakeholder group	N	Age		Gender	
		**Mean**	**Range**	**Men**	**Women**
**Ghent**					
Older adults (experimental group)	4	78	66–90	2	2
Older adults (control group)	5	75	61–88	3	2
Informal carers	6	51.7	36–59	3	3
Volunteers	9	unknown	unknown	0	9
Professionals	4	unknown	unknown	3	1
**Knokke-Heist**					
Older adults (experimental group)	7	75.4	65–87	1	6
Older adults (control group)	4	75.8	73–79	1	3
Informal carers	4	69.8	58–90	2	2
Volunteers	3	68.7	62–80	1	2
Professionals	3	35.7	31–43	1	2
**Tienen**					
Older adults (experimental group)	7	76.3	68–87	4	3
Older adults (control group)	5	70	64–85	1	4
Informal carers	6	62.4	54–67	3	3
Volunteers	5	62.6	57–66	1	4
Professionals	2	56	54–58	0	2

### Intervention

Directly after the first home visit containing the T0 baseline assessment, eligible participants were randomly assigned to either the control or the experimental group by the principal researcher, using computer-generated randomization. Only participants with a middle or high frailty score [11] were included in the intervention part.

In the control group, participants received care as usual which implied that no help or support was mobilized from within the D-SCOPE program. This care as usual was not systematically recorded. In the experimental group, the intervention contained four steps (second home visit, warm referral, the started care/support, follow-up telephone call) in order to empower older adults and improve their access to care and support. These steps are coordinated by a dispatcher. The dispatcher is the pivotal figure between the older adult, the social service of the municipality and the persons who carry out the intervention. He is also the contact person within the municipality/organization.

First, the older participants were contacted by a professional, such as a social worker, from the social service of the municipality for a second home visit. These professionals had experience with conducting home visits and received training and instructions concerning multidimensional frailty, frailty-balance and taking into account the strengths and competences of older adults and their informal caregivers. During the second home visit, the professional from the social service of the municipality further explored the older adult’s competences, needs and preferences. Based on the results of the baseline assessment and on the results of the second home visit, the professional from the social service of the municipality proposed a type of intervention and was responsible for the warm referral. The decision and organization of tailored care and support was made together with the older participant and his/her environment, whereby their competences, strengths and resources were supported. The older participant was accompanied in the referral once decided by which organization and/or form of intervention they could benefit in order to have a higher well-being and/or a lessening of frailty. The started care/support was depending on the availability of the care and support services in the municipality, and could be formal (e.g., home care) or informal (e.g., activities of an older adult’s association) or both. A professional from the social service of the municipality monitored which care/support the participant received, when the older person canceled the care and support and if everything went according to his/her wishes. This monitoring was conducted monthly by a follow-up telephone-call [9].

## Measurements

### Quantitative measurement instruments

#### Socio-demographic and socio-economic characteristics

Specific individual characteristics related to multidimensional frailty were assessed: age (years), gender (man or woman), marital status (married, never been married, divorced, cohabitation, widowed or single), whether the participants had moved in the previous 10 years (yes-no), and country of birth (Belgium - other). Socio-economic characteristics were measured by level of education and monthly household income.

#### Multidimensional Frailty

Multidimensional Frailty was measured using the Comprehensive Frailty Assessment Instrument plus (CFAI+) [5]. For the physical domain, the respondent’s general physical health was assessed (four items, e.g., walking up a hill or stairs. The psychological domain was captured by measuring mood disorders and emotional loneliness (eight items, e.g., losing self-confidence). The social domain was evaluated based on social loneliness (three items, e.g., “There are enough people who I feel close to”) and potential social support network (ten items, e.g., partner, children, and neighbors). Finally, environmental frailty was assessed based on factors related to the suitability of the physical housing environment (five items, e.g., “insufficient comfort in the house”) [12]. Finally, subjective cognitive frailty was assessed based on factors related to cognitive functioning (four items: e.g., memory problems). Scores for each domain, which theoretically range from 0 to 100 [5], were calculated by adding the scores for the specific items. Higher scores indicated more frailty.

#### Well-being

The Short Well-being Instrument for Older adults (SWIO) was used to evaluate three domains of well-being: sense of mastery, meaning in life, and life satisfaction [7]. The SWIO contains a general well-being score (0-100 with higher scores indicating a higher sense of well-being), based on 3 separate dimensions/subscales: Sense of Mastery, Meaning in Life and Life satisfaction. Sense of Mastery (SOM (three items, e.g. “Can’t solve some of the problems that I have”, that measure mastery were derived from the Sense of Mastery scale [13]. Meaning in Life (MIL)—The Dutch version of the “presence” subscale (three items, e.g. “My life has a clear sense of purpose”, of the Meaning in Life Questionnaire [14] was used for the evaluation of meaning in life. Life Satisfaction (LS) (three items, e.g. “I am satisfied with my life”) of the Dutch version of the complete Satisfaction with Life Scale was used [15]. For all measures, responses were given on a five- point Likert scale ranging from 1 (“totally disagree”) to 5 (“totally agree”). A total well-being score can be applied when using the SWIO. The subscales, however, as also applied in this study, can be interpreted separately [7].

#### Satisfaction of D-SCOPE program and intervention

Satisfaction according to the D-SCOPE program and intervention was measured by several questions concerning the degree of satisfaction and meaningfulness of the D-SCOPE methodology. We asked (a) whether the home visits with the questionnaire were useful; (b) if they received a 2nd home visit (of the professional) and if this home visit was meaningful; (c) if they would have preferred a second home visit, in case they didn’t received one; (d) if they were satisfied by the received intervention, and (e) if the home visits, referring to the questionnaires and professional home visits, changed anything in their life. All questions were answered by indicating yes or no. Each time, they had the opportunity to give more information to explain and expand their answer.

### Qualitative interview scheme

The focus groups were designed based on the ‘perceived benefit’ approach [16]. The starting point of this approach is that, in order to assess the effectiveness of an intervention, not only objectively measurable are important; also subjectively experienced effects of the intervention, (hence “perceived benefits”) are interesting to investigate. In the ‘perceived benefit’ approach, the effects of an intervention are judged by the subjective experience of the participant.

The interview scheme consisted of two main topics: (a) perceived benefit or added value of the global D-SCOPE program; (b) experiences, facilitating and hindering components within each of the 6 steps of the D-SCOPE program. A panel of experts approved all the questions, helping to ensure the content validity of the interviews [6]. The expert panel consisted of two neurologists specializing in dementia, a psychologist specializing in neuropsychology and dementia, five adult education scientists specializing in social gerontology, three general practitioners specializing in frailty in later life, and two social gerontologists specializing in public health.

Ten researchers conducted the qualitative semi-structured focus groups. Two researchers were present at each focus group. The researchers received training in conducting focus groups, consisting of four steps: (1) explanation and discussion of the study protocol; (2) explanation and important things to take into account when conducting a focus group; (3) practice conducting focus groups with simulated participants and (4) debriefing on newly identified potential difficulties when conducting focus groups. All the focus groups were held in Dutch and were digitally recorded with Audacity® software (Dominic Mazzoni and Roger Dannenberg, Pittsburgh, Pennsylvania, USA). The focus groups were transcribed verbatim. The head of the research team gave general guidance and specific advice during the data collection period.

### Data analysis

Bivariate analyses, independent sample t-test, χ2-test and Mann–Whitney U test were used to make comparisons of the baseline characteristics between the experimental group and the control group. Paired sample T-tests were carried out to retrieve differences in the experimental and control group’s multidimensional frailty level and well-being at baseline & follow-up. All data were analyzed using SPSS 26.0.

The focus group interviews were analyzed using thematic content analysis, through an adapted version of the Qualitative Analysis Guide of Leuven (QUAGOL) [11]. This analysis comprised two main processes: a thorough preparation of the coding process and an actual coding process. During the preparation of the coding process, each of the ten researchers first read a series of interviews. After discussing these first findings, an initial coding scheme was developed. During the actual coding process, all interviews were divided among the researchers and coded using MAXQDA VERBI Software. All the codes were evaluated and compared, and when necessary, the findings were discussed until consensus was reached.

## Results

### Description participants

**Fig. 1:**
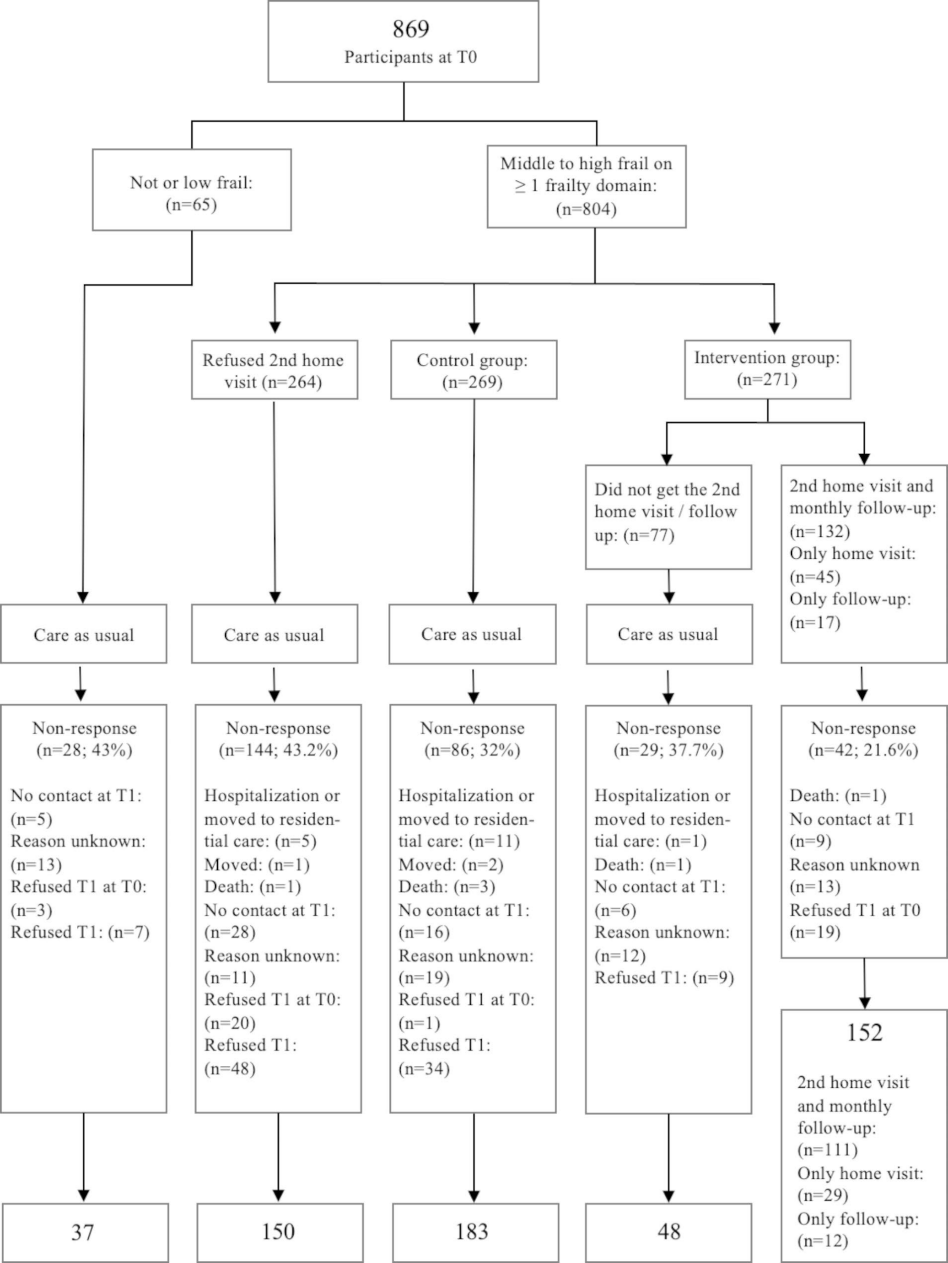
The flow of participants during the program

The flow of participants within the controlled randomized trial is presented in Fig. 1. By means of randomization, participants were allocated to either the control group (n = 269) or the intervention group (n = 271). There were no significant differences in baseline characteristics between both groups (Table 2). During the trial, the dropout rates were 32% (N = 86) in the control and 26% (N = 71) in the intervention group, whereby the main reasons for not participating at T1 were similar in the control and intervention group, i.e., death, no contact or refusal. 77 participants in the intervention group (28%) were not exposed to the intervention-program; they withdrew from a second home visit and a monthly follow-up by telephone, mainly because they did not believe they needed any help.

Table 2 presents the characteristics of all participants at baseline (t0). Mean age was 75.24 years (SD = 8.04) and 49.4% were women. As respondents were selected based on risk profiles for multidimensional frailty, the majority of the sample was divorced (35.4%), followed by being married (31.9%), widowhood (19.2%) and other types for marital status. 47.1% moved in the past ten years and 97.2% had the Belgian nationality. 27.4% respondents had a household income of less than €1250 and 37.8% had a maximum degree of lower secondary education.

Participants who agreed to participate in the intervention were randomized into an intervention group and a control group. An independent sample t-test indicated no differences between the two groups for age (t = − 0.035). χ^2^-tests showed no differences for gender χ^2^ (1, N = 540) = 0.031, p = .860; marital status χ^2^ (1, N = 540) = 2.538, p = .771; moved in the past 10 years χ^2^ (1, N = 540) = 0121, p = .728; or nationality χ^2^ (1, N = 540) = 1.661, p = .436. Mann–Whitney U tests indicated no differences for educational level (Mann–Whitney U = 34,571, p = .478) nor income (Mann–Whitney U = 27298.5, p = .315). For both multidimensional frailty and well-being at T0, independent sample t-tests found no differences between both groups (cognitive frailty t = 1.130; environmental frailty t = 1.608; physical frailty t = 0.318; psychological frailty t = 0.527; social frailty t = − 1.251; meaning in life; t = − 0.803; life satisfaction; t = − 0.912 ; Mastery t = 0.196 ; total well-being; t = − 0.786).

As are the differences between T0 and T1 are concerned, after six months, paired sample t-tests revealed that meaning in life was the only outcome measure to be lower for the control group between baseline (M = 79.00, SD = 19.28) and follow-up (M = 74.06, SD = 20.65); t(450) = 2.295, p = .022. For both the control group as the intervention group, all other measures of multidimensional frailty and well-being did not change significantly (see Table 3).

**Table 2:** Baseline characteristics

	total group		intervention group		control group		
**Demographic characteristics**	N = 869		N = 217		N = 269		
Mean age (SD) ^(1)^	75.24	(8.04)	75.48	(7.66)	75.51	(8.25)	n.s.
No. of women (%) ^(2)^	429	(49.4)	130	(48.0)	127	(47.2)	n.s.
Marital status (%) ^(2)^							n.s.
Married	277	(31.9)	75	(27.7)	81	(30.1)	
Cohabiting	58	(6.7)	16	(5.9)	19	(7.1)	
Widowed	308	(35.4)	94	(34.7)	92	(34.2)	
Divorced	167	(19.2)	64	(23.6)	62	(23.0)	
Never married	57	(6.6)	21	(7.7)	15	(5.6)	
Monastic	2	(0.2)	1	(0.4)	0	(0.0)	
Moved in the past 10 years ( %) ^(2)^	409	(47.1)	133	(49.1)	128	(47.6)	n.s.
Belgian nationality ( %) ^(2)^	845	(97.2)	262	(96.7)	263	(97.8)	n.s.
**Socioeconomic characteristics**							
Education level ( %) ^(3)^							n.s.
None	16	(1.9)	7	(2.6)	5	(1.9)	
Lower education	53	(6.1)	11	(4.1)	25	(9.4)	
Lower secondary education	257	(29.8)	92	(34.3)	82	(30.7)	
Higher secondary education	320	(37.1)	96	(35.8)	98	(36.7)	
Higher university or non-university education	217	(25.1)	62	(35.8)	57	(21.3)	
Income (%) ^(3)^							n.s.
500–999	35	(4.6)	13	(5.5)	11	(4.5)	
1000–1250	172	(22.8)	56	(23.6)	61	(25.1)	
1251–1499	155	(20.5)	58	(24.5)	41	(16.9)	
1500–1999	162	(21.4)	44	18.6	60	24.7	
2000–2499	115	(15.2)	43	18.1	31	12.8	
>2500	117	(15.5)	23	9.7	39	16.0	
**Multidimensional frailty** ^(1)^ **(Mean, SD)**							
Cognitive	22.35	(23.62)	25.98	24.50	23.68	22.19	n.s.
Environmental	11.60	(16.17)	14.70	17.02	12.29	17.45	n.s.
Physical	32.01	(36.45)	37.83	38.66	36.80	36.29	n.s.
Psychological	19.25	(21.59)	23.09	22.20	22.04	23.67	n.s.
Social	47.75	(20.09)	49.32	19.92	51.43	19.03	n.s.
**Well-being** ^(1)^ **(Mean, SD)**							
Meaning in life	76.91	(22.28)	73.85	22.74	75.43	21.86	n.s.
Life Satisfaction	75.78	(21.46)	73.05	23.04	74.81	21.61	n.s.
Mastery	77.11	(25.16)	74.27	25.32	73.85	25.07	n.s.
Total well-being	76.75	(18.69)	73.66	19.28	74.98	18.47	n.s.

Note: n.s. = not significant at the 0.05 level, ^(1)^ = independent sample t-test, ^(2)^= χ2-test, ^(3)^ = Mann–Whitney U test

**Table 3:** Description of respondents’ multidimensional frailty level and well-being at baseline & follow-up

	Intervention group		Control group	
	t0	t1	t0	t1
**Multidimensional frailty** ^(1)^	Mean (SD)	Mean (SD)	Mean (SD)	Mean (SD)
Cognitive	25.15 (23.62)	25.34 (23.36)	23.58 (22.13)	22.25 (20.31)
Environmental	15.63 (16.69)	15.28 (20.33)	10.47 (14.81)	10.33 (14.00)
Physical	38.48 (38.69)	39.57 (38.08)	33.25 (36.26)	33.92 (34.95)
Psychological	23.39 (21.36)	23.18 (21.43)	20.99 (21.81)	19.23 (20.19)
Social	50.27 (19.40)	48.18 (18.88)	48.87 (18.37)	47.64 (19.01)
**Well-being** ^(1)^				
Meaning in life	71.72 (23.81)	73.36 (23.00)	79.00 (19.28)*	74.06 (20.65)*
Life Satisfaction	73.03 (22.92)	72.77 (21.73)	76.94 (20.63)	75.33 (21.46)
Mastery	73.29 (25.12)	72.70 (24.99)	76.17 (24.43)	78.89 (25.06)
Total well-being	72.68 (19.10)	72.94 (18.10)	77.37 (17.33)	76.09 (18.43)

Note: * sig at 0.01 level, ^(1)^ = paired sample t-test

### Satisfaction of D-SCOPE program and intervention

Table 4 summarizes participants’ satisfaction with the program and intervention. 83.9% of the participants indicated that the different home visits within the D-SCOPE program were useful. 27.2% indicated that they received a second home visit of a professional of the Social Centre or municipality and 81.5% indicated that this visit was meaningful. Respondents indicated that this professional home visit was meaningful due to several reasons, e.g., receiving information, having a good conversation, receiving a subvention, knowing their rights and contacts if they need something in the future. Of the respondents who didn’t receive a second home visit, 13.1% preferred one, mostly because they had a need for information. Furthermore, 82.6% were satisfied with the intervention and given information by the professionals during the home visits; 17.6% indicated that the D-SCOPE program changed something to their daily life, e.g., feelings of courage, safety was increased, or they experience more social contact due to the intervention.

In addition to this general evaluation in the questionnaire at T1, the D-SCOPE program was evaluated further in detail in the focus groups. In what follows, every step of the D-SCOPE program is evaluated.

#### Step 1: targeted case-finding

Prior to the intervention, participants were recruited through applying some inclusion criteria. Among the professionals who did the first home visit, there was curiosity whether these risk profiles would result in a more targeted case-finding of frail adults.

“What I find most valuable is actually the fact that those people have already been selected on the basis of those criteria. That, after all these results, I am indeed curious to see whether these criteria could indeed be an important yardstick for us in the future, in order to really look specifically for these people in the neighborhood myself” (Man, professional).

Some of the professionals already experienced more efficient case-finding due to the risk factors, in which they could reach out to older adults who would in most cases not be reached easily or who would not contact the care services on their own initiatives.

“I met a man, I think a man over the age of 85, who wouldn’t have contacted us himself, because he was able to cope with the situation reasonably well, but where we could actually offer a lot in terms of quality and because of which, thanks to the services we have engaged, we can still make it possible for this man to stay at home for a longer period of time, which is his big wish” (Woman, professional).

#### Step 2: home visit 1 by volunteers

Before the home visit of the volunteer took place, a letter, explaining the purpose and proposed time for a visit, was posted to eligible participants. It was an important step according to the older adults, as it was a first introduction to the project. The fact that this letter was send by an organization, which was well-known in some cases, made some older adults more interested and willing to participate. Another advantage of this letter was that people were given time to think about whether or not to participate. Also, in this way informal caregivers could read the letter and explain the project more in depth, or translate it, to the older adults. Some participants indicated that telephonic contact would be more distrusted.

“I have to be honest, if I’d been called on my phone, I would have been suspicious. Because everyone calls for everything. And you don’t know who is on the other side of the phone anymore. Is it reliable? And yes, anyone can say he is from the OCMW (care service), you know? So that letter was important to me. That it is on paper or on mail or whatever. That you see it is okay. That it is official, that is what I think is important. Honestly, when I get a call and someone is at the door, I do not open anymore. No, they first have to put in a note, or I will not open anymore” (Woman, 64 y, control group).

In addition to the letter, the wearing of a badge by the volunteers and the absence of commercial purposes, increased the confidence to participate in the project. Also, different reasons for this participation were raised. Some adults considered the social contact as an advantage, but others realized the advantage of taking part if they would encounter care needs in the future. “Because, it’s becoming clear that help is needed” (Man, 71y, control group).

When doubting to participate, the volunteers tried to convince them about the benefits of the project. When doing this, they did not use ‘healthcare and frailty’ as starting point because they felt some negative connotations with this topic, instead they indicated that the municipality wanted to improve its policy for older people in the future. And, being contacted first by the organization was considered to lower the threshold in access to care, as many indicated to postpone this first step.

“People are scared sometimes, to take that step towards the OCMW. Because people think: the OCMW, that’s an organization for when you are poor. Then you go there. But that is not the case, is it” (Woman, 65y, informal caregiver).

As the first step involved volunteers, keeping them motivated when older adults refused to participate and keeping them convinced of the aim of the project, was experienced as crucial both by volunteers as professionals.

“And yes, I have passed by a lot of addresses for nothing, actually. In the beginning you are still very enthusiastic, but afterwards this was demotivating. At a certain moment I said to myself, I can’t do it anymore, because as a volunteer you also want to get something in return. You must feel that what you are doing is useful’” (Woman, 62y, volunteer).

During home visit 1, the volunteer filled in the survey together with the older adult. In preparation for this, the volunteer received a training where the questions were explained and where they studied the interview scheme in order to anticipate questions. During the focus groups, volunteers stated that it took some practice to conduct the survey in a fluent way. One of the volunteers proposed to lower the threshold by sending a volunteer along with an experienced researcher for the first home visit. Despite a good training, it was especially important to learn by doing. Volunteers gave each other tips when they happened to meet each other in the city or at organized comeback moments with the dispatcher.

“I thought that was very good. For the exchange of ideas from each other, one will explain ‘I’ve done it this way’, the other one had done it that way…. Also, these moments were good for discussing some practical things like those plastic cards [answer cards] so that you there indeed said the questions, I’m going to write down the numbers” (Man, 64y, volunteer).

The visits were seen by the volunteers as highly enjoyable. The informal and spontaneous contact in the personal environment made sure the older person felt comfortable. At the same time, the volunteers got very close to the participants’ personal life during the interview, therefore it was recommended to stress the voluntary character of answering the questions.

“You intrude on the people, too, eh? You, you come very close, don’t you? You ask about their financial side, but also about their state of mind. Most of them are alone, have little or no visitors” (Man, 57y, volunteer).

The contact between the volunteer and the older adult did not always end after the home visit. Some had a drink together in a café, other volunteers also took the initiative to refer the older person to activities in the municipality, such as in the cultural center. These activities were not registered and did not count as official interventions.

Informal caregivers were involved in various ways in the first home visit. They helped for example with the explanation of the information documents or clarification and translation of the questions. However, some informal caregivers wished to be more involved as they felt not informed enough.

“And also what you’ve done… I knew you or someone else was at his home. So I knew that. But every time I asked for it at home: What exactly did these people come to do? Yes, it’s someone from the university. Yes, but what did they come to do? I don’t know. So yes, I don’t really know what you came to do” (Woman, 65y, informal carer).

The older adults appreciated the way in which the survey was conducted. Showing interest in the person’s life was important, but also being professional, empathetic and sympathetic. Pre-printed answer cards were used to answer multiple choice questions. These gave good guidelines according to the participants: “You visualize it that way. It’s clearer and it takes less effort to follow and answer it” (Man, 75y, control group).

#### Step 3: Professional home visit

The professionals experienced two main advantages of the preparatory work of the volunteers in step 2. First, professionals expressed it was easier for them to enter the houses of the participants for a second home visit, because the volunteers had paved the path in step 2. Second, by using the results of the first survey (administered in step 2), the professionals felt sufficiently prepared for the home visits. Building on data from the different frailty dimensions, they could prepare their visit more targeted. For example, during the professional home visits, special attention was given to exchanging (targeted) information on the different services existing in the municipalities. This was an important first step to actual care and support, since many participants were not aware of the offer. The social contact in this process of information exchange was crucial for the participants.

During this second home visit, various intervention options were discussed, looking beyond solely medical and classical home care. Other interventions were for instance: referral to hot meals in the community center, enrolment in a neighborhood-buddy project or a new course, etc.

The professionals however highlighted that they felt misinformed in the beginning. They expected to detect more severe frail older adults than they actually did. Only after some time in the program they realized the benefits of this approach, and the need to make a ‘shift in mind from intervention to prevention’. In responding question what they experienced as a benefit one of the professionals said:

“And also, this preventive aspect, because that was in the past less important to us. We mainly went to the oldest one’s. And now we have gone to people aged between 60 and 70 and that is a group that we initially thought of ‘as being okay’. And also, after those first home visits, we taught we can’t really offer any added value or direct help, which is what we actually do in our job are what we used to. But then, as the project progressed, yes, it started to dawn this is actually very, very important that we also preventively do something and that people know that they can come to us from the moment they experience difficulties” (Woman, professional).

In that line of reasoning some older adults suggested that people should receive such a first home visit around their retirement. Even more, several different stakeholders expressed this home visit as a form of intervention. Even when no care or support was started, professionals informed and explained the available services and activities, which was considered to be an intervention.

“… She (an older woman) had a number of questions, she didn’t know the local service center. Ah, you have a handyman, how does that work? So actually, in her case I did perform an intervention, so to speak, because I gave her the information she asked for at that time and I think, within a year or two, if she really becomes less mobile, we will see her again anyway. So, I do regard this case as an intervention. But I haven’t started any referrals or anything. But the people know us now” (Man, professional).

#### Step 4: ‘Warm referrals’

Instead of merely giving a service name or number (i.e., cold referral), a warm referral indicates that the participants received more intensive support in contacting the appropriate care and support. As this older woman explains:

“V (name) also took me to the town hall because she knew a service there where I could get an allowance. And she sorted this for me right away” (Woman, 65y, experimental group).

Respondents stated that crucial elements for a high-quality warm referral were the speed of contacts after the home visit, the constancy of contacts and the “approaching work” of the professional. In addition, the commitment and directness of how healthcare providers set to work was highly appreciated by the older adults in the experimental group. Reaching a shared agreement and understanding was important for the satisfaction and involvement of older adults.

“I was very surprised. Maybe a week or two later, I received the first phone call, ‘I’ve heard through the survey that you would like to have this and that’. They (local service center) responded very quickly. And there was constant contact, telephone contacts for several reasons for example that she had found something that might be of interest to me. And I must admit, everyone was very friendly, really well-done. I don’t regret taking part in the survey” (Woman, 65y, experimental group).

Not all home visits resulted into an immediate intervention, because this was not always necessary. Professionals brought along information that participants could reread afterwards. However, this was also perceived as a ‘warm referral’, when older adults were guided through the brochures, and when the content of the brochures was clearly explained. Respondents highlighted that this approach increased the possibility that older adults would use this information in the future. As a form of prevention this was valued by both professionals, informal caregivers as older adults.

#### Step 5: implementation of the intervention

A wide range of interventions was carried out. Both small-scaled care or support (e.g. parking disc for older people with mobility issues or filling in administration) as larger interventions (e.g. implementing weekly home care services) were recognized as important for older clients. In addition, these interventions were not only designed to prevent or delay frailty, but rather to increase the well-being of the older person. Based on their wishes (irrespective of their ‘frailty’) actions were designed and valued. As a professional points out:

“We had an alarm installed there, because he was an unmarried and living alone. He had no informal care and lived in a house with lots of stairs. He did not have a cell phone and the phone was downstairs and he usually sat in the porch, so he had to take the stairs every time to pick up the phone. In the meantime, he also had meals delivered at home, because he only occasionally made meals, but that was mostly soup (…) We could think about so many things we could do for these men… But: he had a lot of videos of the travels he had made in the past, but he couldn’t find a video player anymore. Now it is DVD players everywhere. And we really searched this for him, we found one and actually this meant so much for that man… But we also felt that this generated a lot more confidence from that man in our service. This little something actually made it possible for us to do a lot in terms of trust. I think if that man has something in mind, that he will contact us now” (Woman, professional).

#### Step 6: aftercare through follow-up calls

Because we anticipated a risk of dropping-out, the 6th step of the D-SCOPE project concerned monthly follow-up calls (1month after home visit 2, 2 months, 3 months, 4 months). Calls were performed by the dispatcher who was well-informed about the situation of the respondent. One of the professionals from the social service of the municipality kept a notebook with some detailed information, so she could check whether there were changes in the situation when calling back. People who are vulnerable sometimes do not realize that their problems may persist for a longer period. Admitting that they needed help, seemed difficult. However, when performing regular phone calls by the same professional increased the chance of asking for help, because older people gained enough confidence, or something had happened that changed their situation.

“… there are a lot of people who refuse help, or they don’t need it now. But sometimes by continuing to call or continue to go to them, at once they will tell that something radical has happened, or that they are lonely” (Woman, professional).

When people asked to not be called again in the future, the professional from the social service of the municipality checked and recorded the reasons for this drop-out (e.g., dissatisfied clients, care avoiders, etc.). The follow-up calls were recognized as one of the greatest strengths of the project by both older adults, their informal care takers, and the professionals. However, these calls were seen as time-consuming, despite the obvious added value. The professionals indicated that a monthly follow-up was too frequent for most people, but that it should be personalized according to the needs of the person. Also, informal caregivers expressed their appreciation about this follow-up call and even asked to have follow-up calls with them.

**Table 4:** Overview satisfaction of D-SCOPE program and intervention

	N	% Yes
Home visits with the questionnaire were useful.	552	83.9
They did not receive a second home visit (professional) but would have liked to have one.	406	13.1
Home visit (professional) was meaningful.	146	81.5
Satisfied with the care, support, or information due to home visits.	167	82.6
Home visits (referring to the surveys and professional home visits) have led to significant changes in their lives.	307	17.6
Note: The number of observations is variable due to missing values.		

## Discussion

This study presents an overview of a mixed-methods study within the larger D-SCOPE project, in which the results of quantitative and qualitative analyses shed light on the process of the developed 6-step model. This solid 6-step model is used for more efficient preventive home visits. Until now, various municipalities and organizations are setting up preventive home visits to see what the needs of the older adults are. However, this is often staff-intensive and time-consuming. In order to make these home visits more efficient and effective, the first step of the model aims at detecting frail older adults based on specific risk profiles for frailty (a combination of gender, age, marital status and having moved). Professionals experienced that working with risk profiles led to a more efficient case-finding. In accordance with the present results, a more recent study also indicates that the D-SCOPE risk profiles detect more frailty [17]. As a result, these home visits can be much more targeted in the future towards frail older people.

Second, the results demonstrate that a ‘mind-shift’ in care from intervention to prevention is needed. Both the government and the older adults themselves prefer to live at home as long as possible. In order to make this possible, care and support will have to focus more on prevention rather than mainly on intervention. This requires early detection of frail older people [9]. It will give us insights into older people who are struggling and who are in danger of becoming frail (or already are) in their home situation. Thanks to early detection and the setting up of early interventions, a lot of problems can already be prevented. These interventions do not have to be big, also ‘small help’ is valuable and crucial. The importance of prevention can also be seen in light of the result that on prevention level, 82.6% of the respondents were satisfied with the intervention and given information. While on intervention level, 17.6% indicated that the D-SCOPE program changed something to their daily life. A review study on interventions for frail older adults already pointed out that no significant effects are found after interventions [17]. This study shows that the success of an intervention can be found in small positive results. The cost-effectiveness of D-SCOPE is not discussed in this paper. The focus was mainly on prevention and preventive methods are not necessarily cost-effective. If setting up a preventive intervention is more expensive than doing nothing, this is not necessarily a reason not to implement the preventive methods. After all, these interventions are often an investment in the well-being of current and future generations and can therefore have an impact on the population as a whole. Preventive interventions may therefore not be cost-effective, but they are effective and therefore worth implementing [18–21].

There is also a need for a broad view on care and support services. The D-SCOPE project shows that care and support must go beyond the organization’s own offer. The starting point for this is the individual wishes and needs of a person who’s seeking help. As research already pointed out that needs for care, support and empowerment is highly personal [22]. Cooperation between different care and service providers, across the organizations, will contribute to the well-being of the older adults. An important success factor in this regard is that the dispatcher works independently of an organization.

The results of the focus groups demonstrate the added value of recognizing frail older adults in their strengths and starting the care and support discussion oriented at their well-being, and not at reducing or delaying frailty. Frailty is often viewed from a negative perspective, with a focus on what people can no longer do [8]. That is why many projects work to reduce frailty and eliminate ‘deficits’, very often resulting in finding no effectiveness of interventions [23]. D-SCOPE shifts this dominant, negative focus to a positive approach focused on the strengths and competences of older people and their immediate environment, despite their frailty. Research shows that it is more important to focus on improving the well-being of frail older people. For example, 23.1% of older people who are seriously vulnerable in at least one area still experience a very good quality of life. This is in line with others studies who found an important number of frail older people with a good quality of life [24–26]. In order to be able to respond to the increase in well-being, despite frailty, it is important to examine per individual which strengths, capacities and/or resources contribute (or can contribute) to this well-being. These “balancing factors” (strengths, capacities, and resources) form the entry point for interventions. Through this empowerment approach, older adults can be encouraged to participate in care decisions and actions that improve their quality of life [27, 28].

Finally, a need for organizations to work outreaching is seen. If care and welfare organizations become familiar faces in the neighborhood, even for those who do not yet need help, the barrier to formal care could be lowered [29]. This also includes ‘warm referral’, to lead the older adult to the appropriate care. Moreover, the D-SCOPE project showed the importance of regular contact and accessible follow-up telephones after a preventive home visit. Their importance was already showed to prevent hospital readmissions after hospital discharge [30, 31]. At the moment of a preventive home visit, it was possible that no care needs were expressed because the trust in an organization was still too low. Through the follow-up telephone, a trust is built up, so that after one, two, or three conversations care was still started. As Crocker et al. [32] explains, patients’ engagement with their primary care providers is positively influenced by follow-up telephones. Additionally, the chance of drop-out after the start of the care is real, a follow-up telephone will overcome this.

## Limitations

Our findings should be considered in light of the following limitations. First, our follow-up assessment was conducted six months after step 2 (preventive home visit by the volunteer). This may hamper comparisons with other studies that apply a more common follow-up period of 12 or 24 months. Nevertheless, already after 6 months the drop-out was considerable: 34.4%. In this study, the two home visits were sometimes following short after each other and requested a big engagement of participants, this could possibly be a reason for dropout of some respondents. Second, even though the participating coordinators from the municipality received extensive training and guidance (e.g. one day-training at the start, 5 intervision days, and a personal contact person at the university for questions and support), and it was designed as a train-the-trainer package, the shifts in mind “from intervention to prevention” and “from deficit to strengths” were not always easily translated to their colleagues and professionals who performed the home visits. In the future more attention should be devoted to training the professionals training the home visits themselves, directly, not via train-the-trainer. Finally, the process of implementing the D-SCOPE program differed in the three municipalities, in terms of success in recruitment of volunteers, timing between home visit 1 and home visit 2, and type of care and support installed. Important influencing contextual factors are: enthusiasm and engagement of the coordinator in the municipality, support of management and local government, local supply of services and projects, and local experience with innovative projects.

## Conclusion

This study aimed to give an overview of the descriptive results of the D-SCOPE program and evaluate the process. Our quantitative data show that the vast majority of participants found the home visits meaningful and were satisfied with the intervention. The qualitative findings provided more insights into the experiences of the participants with the process. Based on these insights of the 6-step model of preventive home visits, municipalities and organizations can apply this model to carry out more targeted home visits. Therefore, a mind-shift from intervention to prevention is needed, as well as an open view on the broad range of care and support services. These services can also be seen from a strengths-based approach, rather than only a deficit-based approach. These frail older adults can best be reached through an outreach approach with ‘warm referrals’, to lower the barriers to formal care services.

## Data Availability

The datasets used and analyzed during the current study are available from the corresponding author on reasonable request.

## Declarations

### Consortium name

Full list of consortium members and affiliations Smetcoren, An-Sofie ^1^ Dury, Sarah ^1^ De Donder, Liesbeth ^1^ Dierckx, Eva ^1,2^ Lambotte, Deborah ^1^ FRET, Bram ^1^ Duppen, Daan ^1^ Switsers, Lise ^1,3^ Hoens, Sylvia ^1^ Kardol, Martinus ^1^ De Witte, Nico ^1,4^ VertÉ, Dominique ^1^ Hoeyberghs, Lieve ^4^ De Roeck, Ellen ^5^ Engelborghs, Sebastiaan ^5^ Dedeyn, Peter Paul ^5^ Van der Elst, Michael ^6^ De Lepeleire, Jan ^6^ Schoenmakers, Birgitte ^6^ Van der Vorst, Anne ^7^ Zijlstra, G.A. Rixt ^7^ Kempen, Gertrudis I.JM. ^7^ Schols, Jos M.G.A. ^7 1^ Faculty of Psychology and Educational Sciences, Vrije Universiteit Brussel(VUB), Brussels, Belgium ^2^ Psychiatric Hospital Alexianen Zorggroep Tienen, Tienen, Belgium ^3^ Research Foundation Flanders (FWO), Brussels, Belgium ^4^ Faculty of Education, Health and Social Work, University College Ghent, Ghent, Belgium ^5^ Laboratory of Neurochemistry and Behavior, University of Antwerp, Antwerp, Belgium ^6^ Department of Public Health and Primary Care, University of Leuven, Leuven, Belgium ^7^ Department of Health Services Research, Care and Public Health Research Institute (CAPHRI), Maastricht University, The Netherlands.

## List of abbreviations

D-SCOPE: Detection, Support and Care for Older people, Prevention and Empowerment.
SWIO: Short Well-being Instrument for Older adults.
QUAGOL: Qualitative Analysis Guide of Leuven.
